# Re‐irradiation of recurrent gliomas: pooled analysis and validation of an established prognostic score—report of the Radiation Oncology Group (ROG) of the German Cancer Consortium (DKTK)

**DOI:** 10.1002/cam4.1425

**Published:** 2018-03-23

**Authors:** Stephanie E. Combs, Maximilian Niyazi, Sebastian Adeberg, Nina Bougatf, David Kaul, Daniel F. Fleischmann, Arne Gruen, Emmanouil Fokas, Claus M. Rödel, Franziska Eckert, Frank Paulsen, Oliver Oehlke, Anca‐Ligia Grosu, Annekatrin Seidlitz, Annika Lattermann, Mechthild Krause, Michael Baumann, Maja Guberina, Martin Stuschke, Volker Budach, Claus Belka, Jürgen Debus, Kerstin A. Kessel

**Affiliations:** ^1^ Department of Radiation Oncology Technical University Munich (TUM) Munich Germany; ^2^ Institute of Innovative Radiotherapy (iRT) Helmholtz Zentrum München Neuherberg Germany; ^3^ Partner sites Munich, Heidelberg, Berlin, Frankfurt, Tübingen, Freiburg, Dresden, Essen German Cancer Consortium (DKTK) Berlin Germany; ^4^ Department of Radiation Oncology University Hospital LMU Munich Munich Germany; ^5^ Department of Radiation Oncology Heidelberg Institute for Radiation Oncology (HIRO) Heidelberg University Heidelberg Germany; ^6^ Department of Radiation Oncology Charité‐University Hospital Berlin Berlin Germany; ^7^ Department of Radiation Oncology University Hospital Johann Wolfgang Goethe University Frankfurt Germany; ^8^ Department of Radiation Oncology Faculty of Medicine University Hospital Tübingen Eberhard Karls University Tübingen Tübingen Germany; ^9^ Department of Radiation Oncology University Medical Center Freiburg Freiburg Germany; ^10^ Department of Radiation Oncology and OncoRay National Center for Radiation Research in Oncology (NCRO) Faculty of Medicine University Hospital Carl Gustav Carus Technische Universität Dresden Dresden Germany; ^11^ Institute of Radiooncology Helmholtz‐Zentrum Dresden‐Rossendorf Dresden Germany; ^12^ Partner site Dresden National Center for Tumor Diseases (NCT) Dresden Germany; ^13^ Deutsches Krebsforschungszentrum (DKFZ) Heidelberg Germany; ^14^ Department of Radiotherapy University Hospital Essen University of Duisburg‐Essen Essen Germany

**Keywords:** Outcome, prognostic score, recurrent glioma, re‐irradiation

## Abstract

The heterogeneity of high‐grade glioma recurrences remains an ongoing challenge for the interdisciplinary neurooncology team. Response to re‐irradiation (re‐RT) is heterogeneous, and survival data depend on prognostic factors such as tumor volume, primary histology, age, the possibility of reresection, or time between primary diagnosis and initial RT and re‐RT. In the present pooled analysis, we gathered data from radiooncology centers of the DKTK Consortium and used it to validate the established prognostic score by Combs et al. and its modification by Kessel et al. Data consisted of a large independent, multicenter cohort of 565 high‐grade glioma patients treated with re‐RT from 1997 to 2016 and a median dose of 36 Gy. Primary RT was between 1986 and 2015 with a median dose of 60 Gy. Median age was 54 years; median follow‐up was 7.1 months. Median OS after re‐RT was 7.5, 9.5, and 13.8 months for WHO IV, III, and I/II gliomas, respectively. All six prognostic factors were tested for their significance on OS. Aside from the time from primary RT to re‐RT (*P* = 0.074) and the reresection status (*P* = 0.101), all factors (primary histology, age, KPS, and tumor volume) were significant. Both the original and new score showed a highly significant influence on survival with *P* < 0.001. Both prognostic scores successfully predict survival after re‐RT and can easily be applied in the routine clinical workflow. Now, further prognostic features need to be found to even improve treatment decisions regarding neurooncological interventions for recurrent glioma patients.

## Introduction

The heterogeneity of glioma recurrences remains an ongoing challenge for the interdisciplinary neurooncology team. The value of neurosurgical resection has been shown by several authors 1, 2, 3, 4, 5, 6. Novel multicenter data have demonstrated that the resection of glioma recurrences is a prognostic factor, regardless of the extent of resection 7. Nearly, all patients with recurrent gliomas have been treated with radiotherapy (RT) after primary diagnosis. Therefore, a second irradiation was initially prescribed cautiously due to the fear of unwanted effects 8. With increasing experience and knowledge of healthy tissue toxicity profiles, in line with the continuous improvement of RT regarding precision, re‐irradiation (re‐RT) has now been established within clinical routine 9, 10, 11, 12, 13.

Response to re‐RT is heterogeneous, and survival data depend on prognostic factors such as tumor volume, primary histology, age, the possibility of reresection, or time between primary diagnosis and initial RT and re‐RT 14, 15. In the past, selection criteria for re‐RT have been established, most of which depend on institutional guidelines, personal preferences, and/or historically developed recommendations: Generally, re‐RT is only applied in patients with macroscopic tumors, at least 6 months after initial RT, and with a tumor diameter up to 4 cm 16. All other factors are in general not taken into account. In the past, we analyzed patients with recurrent high‐grade gliomas treated for re‐RT and developed a prognostic score for outcome 14, 15. While some groups discussed the score critically, an independent cohort validated the approach several years after that 17, 18, 19. However, there is an ongoing controversy about the real prognostic factors, and which scoring system is the best tool for decision making in clinical practice.

In the present pooled analysis, we gathered data from nine large German radiooncology centers of the German Cancer Consortium—Radiation Oncology group DKTK‐ROG (Deutsches Konsorium für Tranlationale Forschung, DKTK). This large independent, multicenter cohort of 565 patients was used to determine the outcome after re‐RT and to validate the established prognostic score by Combs et al. 14 and its modification 15.

## Methods

### Patients and treatment

The DKTK‐ROG database 20 provided 565 recurrent high‐grade glioma patients treated with re‐RT from 1997 to 2016. Each site chose patients randomly from their local data and documented the clinical information retrospectively (Table S1). Inclusion criteria were age >18 years and a histology of a high‐grade glioma at re‐RT. Primary histology changed in 60 cases to a higher grade at the time of recurrence (47 cases to WHO IV; 13 cases to WHO III). This was confirmed by either biopsy or reresection (*n* = 108). For patient characteristics, see Table 1. The local ethics committee of each site approved the study.

**Table 1:** Patient characteristics

	All patients, (*n* = 565), *n* (%)	Validation cohort (*n* = 356), *n* (%)
Gender		
Female	213 (37.4)	134 (37.6)
Male	352 (62.3)	222 (62.4)
Age at re‐RT (median, range) [years]	54 (18–82)	54 (18–76)
≥50	354 (62.7)	234 (65.7)
<50	211 (37.3)	122 (34.3)
Primary histology at diagnosis		
WHO IV glioma	454 (80.4)	307 (86.2)
WHO III glioma	82 (14.5)	44 (12.4)
WHO I/II glioma	18 (3.2)	5 (1.4)
Unknown	11 (1.9)	
Histology at re‐RT		
WHO IV glioma	500 (88.5)	325 (91.3)
WHO III glioma	65 (11.5)	31 (8.7)
KPS at re‐RT		
<80%	298 (52.7)	135 (37.9)
≥80%	228 (40.4)	221 (62.1)
Unknown	39 (6.9)	
Tumor volume (PTV) at re‐RT (median, range) [mL]	54.4 (0.4–489.0)	67.6 (0.4–489.0)
≤47 mL	217 (38.4)	144 (40.4)
>47 mL	292 (51.7)	212 (59.6)
Unknown	56 (9.9)	
Reresection		
Yes	108 (19.1)	94 (26.4)
No	301 (53.3)	262 (73.6)
Unknown	156 (27.6)	
Time from primary RT to re‐RT, (median, range) [months]		
WHO IV glioma	12.9 (1.8–173.8)	13.2 (1.8–136.9)
WHO III glioma	32.1 (0.8–198.7)	34.3 (4.6–176.8)
WHO I/II glioma	45.2 (11.2–265.3)	84.1 (39.0–265.3)
MGMT status		
Methylated	135 (23.9)	112 (31.5)
Not methylated	161 (28.5)	129 (36.2)
Unknown	269 (47.6)	115 (32.3)
re‐RT dose		
Radiosurgery (only WHO IV)	15 (12–21)	15 (12–21)
re‐RT dose per fraction	2.67 (1.2–6.25)	2 (1.6–6)
re‐RT total dose	36 (20–70)	36 (20–60)
Concurrent chemotherapy		
Yes	315 (55.8)	235 (66.0)
No	130 (23.0)	110 (30.9)
Unknown	120 (21.2)	11 (3.1)

re‐RT, re‐irradiation; KPS, Karnofsky Performance Score; PTV, Planning target volume; MGMT, O6‐Methylguanin‐DNA‐Methyltransferase.

For most patients, a median dose of 36 Gy was applied as fractionated re‐RT and in 11 cases with a median of 15 Gy as radiosurgery. Primary RT was between 1986 and 2015 with a median dose of 60 Gy (range 45–66 Gy, single dose 1.2–3.0 Gy) using different techniques such as 3D or intensity‐modulated radiotherapy (IMRT). Treatment planning and follow‐up procedures followed the individual institutional guidelines.

### Score calculations

For the original score by Combs et al. 14, one determines the prognostic value of the factors: primary histology, time from primary RT to re‐RT, and age (Table 2). The sum of these three values is the final score; values from 0 to 4 are possible. For the new score by Kessel et al. 15, one also adds values for Karnofsky Performance Score (KPS), tumor volume (PTV, planning target volume), and performed reresection. The sum is a value from 0 to 7. For the final new score, there are four scoring groups with the following values: *a* = 0–1, *b* = 2–3, *c* = 4–5, *d* = 6–7.

**Table 2:** Scoring scheme of the original 14 and new 15 score

Prognostic factor	Prognostic value of the original score	Prognostic value of the new score
Primary histology at diagnosis		
WHO IV	2	2
WHO III	1	1
WHO I/II	0	0
Age		
≥50 years	1	1
<50 years	0	0
Time from primary RT to re‐RT		
≤12 months	1	1
>12 months	0	0
KPS		
<80%		1
≥80%		0
Tumor volume (PTV)		
>47 mL		1
≤47 mL		0
Reresection performed		
No		1
Yes		0

re‐RT, re‐irradiation; KPS, Karnofsky Performance Score; PTV, Planning target volume.

### Score validations

We validated both scores: (1) the original score including the three prognostic factors: initial histology, age, and time from primary RT to re‐RT; (2) the modified new score with the additional factors KPS, PTV, and performed reresection (Table 2). The total number of cases from all centers was 565. Due to missing parameters in the data pool, we excluded incompletely documented patients. We calculated the original score with data from 552 cases and the new score with 356 cases.

### Statistics

Overall survival (OS) was calculated from the first day of re‐RT until death or last follow‐up based on the Kaplan–Meier method. We performed univariate and multivariate analyses using Cox proportional hazards regression. Significance was determined by a needed level of *P* ≤ 0.05. All statistics were performed using SPSS v23 (IBM, New York, NY).

## Results

### Survival analyses

Median age was 54 years (range 18–82 years); median follow‐up was 7.1 months (95% CI: 9.2–11.4 months). Median OS after re‐RT was 7.5 (95% CI: 6.7–8.3 months), 9.5 months (95% CI: 6.3–12.7 months), and 13.8 months (95% CI: 12.4–15.2 months) for initial WHO IV, III, and I/II gliomas, respectively.

All six prognostic factors were tested for their significance on OS using univariate and multivariate analyses. Aside from the time from primary RT to re‐RT (*P* = 0.076) and the reresection status (*P* = 0.101), all factors (primary histology, age, KPS, and tumor volume) were significant in the univariate model (Table 3). Only age and KPS remained significant in the multivariate analysis. MGMT status (O6‐Methylguanin‐DNA‐Methyltransferase) was available in about half of the cohort. In these patients, it showed a significant influence on survival (*P* = 0.002).

**Table 3:** OS analysis of the prognostic factors and scores

Prognostic factor	Univariate analysis			Multivariate analysis		
	HR	95% CI	*P*‐Value	HR	95% CI	*P*‐Value
Primary histology at diagnosis	1.28	1.06–1.54	0.010*	1.29	0.98–1.69	0.069
Age (≥50 y vs. <50 y)	1.45	1.21–1.75	<0.001*	1.35	1.06–1.73	0.015*
Time from primary RT to re‐RT (≤12 m vs. >12 m)	1.18	0.98–1.41	0.074	1.09	0.86–1.38	0.486
KPS (<80% vs. ≥80%)	2.02	1.67–2.43	<0.001*	1.80	1.41–2.29	<0.001*
Tumor volume (PTV) (>47 mL vs. ≤47 mL)	1.23	1.02–1.49	0.032*	1.26	1.00–1.60	0.056
Reresection performed (no vs. yes)	0.82	0.65–1.04	0.101	0.80	0.62–1.04	0.099
MGMT status (methylated vs. not methylated)**	0.67	0.52–0.86	0.002*	–	–	–
Score						
Original score	1.20	1.10–1.32	<0.001*	–	–	–
New score	1.22	1.11–1.34	<0.001*	–	–	–

*significant *P*‐value; **no prognostic factor for the score calculations; *n*, number of cases for which the prognostic factor was documented; KPS, Karnofsky Performance Score; PTV, Planning target volume; re‐RT, re‐irradiation; y, years; m, months.

### Score validation

**Figure 1:**
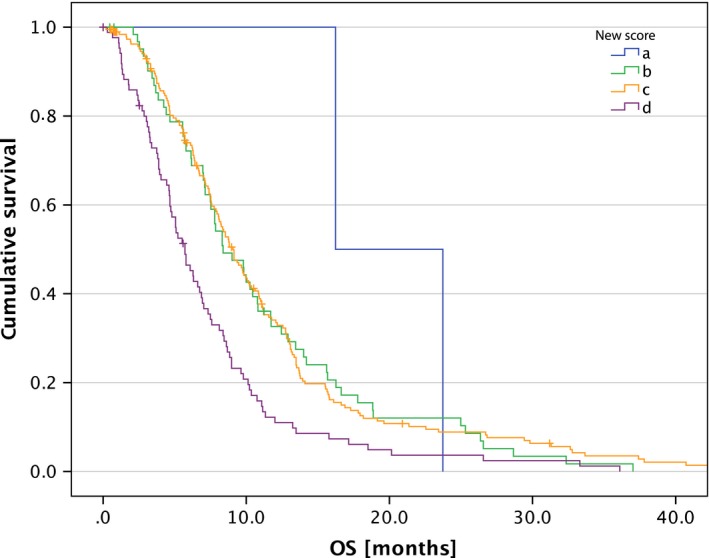
OS after re‐RT according to the original score (*P* < 0.001).

**Figure 2:**
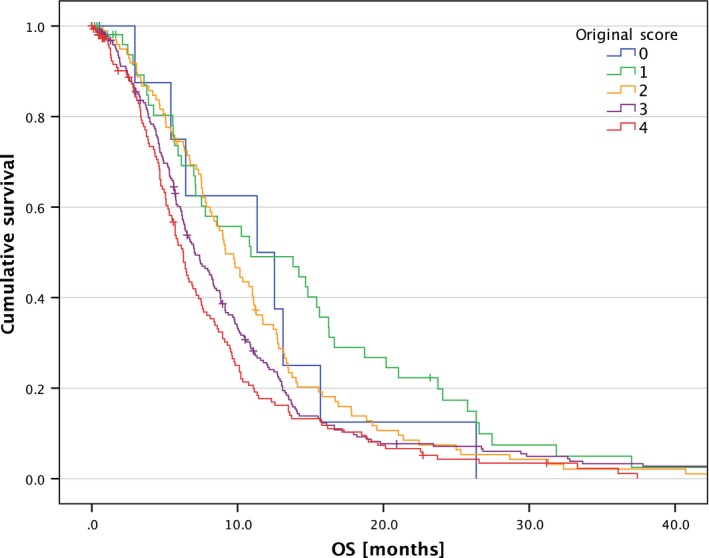
OS after re‐RT according to the new score (*P* < 0.001).

We calculated the original score by Combs et al. 14 and the new score by Kessel et al. 15 according to the calculation scheme described in Table 2. The original score was calculated with 552 cases and showed a significance with *P* < 0.001 (Fig. 1); the new score was calculated with 356 cases and was highly significant with *P* < 0.001 (Fig. 2). Table 4 lists median OS and life tables for both scores.

**Table 4:** Median OS and life table for both scores

	*n*	Median OS	Proportion surviving after re‐RT			
			6 months (%)	12 months (%)	24 months (%)	36 months (%)
Original score						
0	9 (2%)	12.0	75	50	13	0
1	60 (11%)	11.3	76	49	22	5
2	106 (19%)	9.7	76	35	8	2
3	224 (41%)	7.5	64	26	8	3
4	153 (28%)	6.6	57	18	5	2
New score						
a	2 (1%)	16.8	100	100	50	0
b	67 (19%)	9.4	75	34	12	2
c	199 (56%)	9.4	75	34	10	4
d	88 (25%)	6.1	50	12	4	1

OS, Overall survival; *n*, number of patients.

## Discussion

The study aimed to validate the prognostic score by Combs et al. 14 and its modification by Kessel et al. 15 based on a large independent, multicenter cohort of 565 patients. This is the largest validation cohort existing. The data demonstrated nicely a highly significant correlation between the scores and OS after re‐RT (original score: *P* < 0.001; new score: *P* < 0.001).

Second radiation therapy has become an accepted treatment pillar in the multidisciplinary canon of treatments for recurrent gliomas. Today, with stereotactic radiotherapy (SRT) and other highly conformal options, as well as improved target volume definition by more elaborate imaging, the improvement in precision correlated with a reduction in side effects and a possible increase in dose 21, 22. Thus, re‐RT is now used more widely. Hypofractionated approaches (HSRT) up to a single dose of 7 Gy and total dose up to 42 Gy showed excellent OS of median 7.4–12.7 months 12, 23, 24, 25, 26. In comparison, our cohort showed an OS of 9.2 and 7.4 months for WHO III and IV patients, respectively. Clarke et al. 27 found in their recent phase 1 study with concurrent bevacizumab and HSRT for high‐grade gliomas doses of 33 Gy in 3 × 11 Gy fractions acceptable and well tolerated with an OS of 13 months. However, there is no standard treatment regarding fractionation, dose or time between RTs, and institutional preferences differ enormously 28. Hence, a score including the significant prognostic parameters is a helpful decision‐making tool.

Previous attempts to validate the original score failed 19, 29. Several reasons might be discussed for this effect: small cohorts, missing data relevant to evaluation, different re‐RT in‐house standards. With a patient cohort from our institution only (*n* = 199), we recently validated the initial score and could show that significance remains strong also in an independent patient group (*P* < 0.001) 18. We then modified the initial score as we could demonstrate that other factors are also highly relevant for outcome 7, 30, 31, 32 and added tumor volume, KPS, and reresection status to the initial approach. In the present analyses, we could demonstrate the significance of KPS (*P* < 0.001) and tumor volume (*P* = 0.032) and in particular the reliability of the modified new score (*P* < 0.001) (Table 3). The factors resection (*P* = 0.101) and time from primary to re‐RT (*P* = 0.074) were not significant. That might be attributed to the heterogeneity of the data and different treatment regimens in this present multicenter cohort. However, this analysis aimed to validate an existing score and use the calculation scheme as is. We think it is a highly relevant finding that both scores remain significant in such large groups of patients, reproducibly. This is highly relevant and stresses that perhaps this score can be useful for clinical decision making in the future.

One of the main advantages of our score is its simple way of calculation. It is easy to understand and can be applied in the routine workflow or evaluation procedures of any other clinic. Only six variables (primary histology, age, KPS, tumor volume, time from primary RT to re‐RT, reresection status) are needed that are usually available for every case. Most patients decide for additional treatment regardless of the risks and benefits. Before treatment decision of re‐RT, the score could be calculated as one further decision‐making tool, and the physician could use the result to counsel the patient most effectively. Even before reresection, it can help to determine the potential risk and benefit for the recurrent glioma patient. One could propose that for a patient with score “d” and a median OS of 5.7 months, a hypofractionated approach should be prescribed as it is a short treatment and the patient is not spending several weeks in the hospital during RT.

Our analyses have several limitations. Apart from the missing values from some sites, multicenter studies contain heterogenic data regarding treatment and data documentation. Known prognostic factors such as MGMT status and IDH mutations are not included in the scores. These parameters are still not determined by default in routine treatment and could even change during the disease. In particular, the current MGMT status is rarely available before re‐RT. In the multivariate analyses, only age and KPS remained significant. This reflects perhaps best daily clinical practice. However, the scores remained highly significant and therefore are potentially relevant for further patient stratification.

In the era of personalized and individualized medicine, more factors will be included, and many research groups are working on prognostic features to prescribe the best therapy possible. Recently, also radiomic features have been researched to develop another image‐based score mechanism 33, 34, 35. Features such as tumor location, shape, and gray level might be relevant for image‐based analyses to predict prognosis.

## Conclusion

This is the largest cohort to validate the prognostic scores published previously. Both prognostic scores by Combs et al. 14 and Kessel et al. 15 successfully predict survival after re‐RT. Both scores are easy to apply and thus practical to include into treatment decision making. Further prognostic features might improve treatment decisions regarding neurooncological interventions for recurrent glioma patients.

## Conflict of Interest

None declared.

## Supporting information


**Table S1.** Patient distribution according to the participating site.
