# Supplementary material for: Re‐irradiation of recurrent gliomas: pooled analysis and validation of an established prognostic score—report of the Radiation Oncology Group (ROG) of the German Cancer Consortium (DKTK)
Source: Cancer Med. 2018 Mar 23;7(5):1742–9. doi: 10.1002/cam4.1425 (PMC5943421; doi:10.1002/cam4.1425)
Supplement: Supplementary file 1 — Table S1. Patient distribution according to the participating site. [file CAM4-7-1742-s001.docx]

Supplement

Table 1: Patient distribution according to the participating site.

| Site | Number of patients | Number of patients used for validation of the original score | Number of patients used for validation of the new score |
| --- | --- | --- | --- |
| All | 565 | 552 | 356 |
| Berlin | 139 | 126 | 27 |
| Dresden | 28 | 28 | 26 |
| Essen | 17 | 17 | 17 |
| Frankfurt | 52 | 52 | 48 |
| Freiburg | 33 | 33 | 0 |
| Heidelberg | 108 | 108 | 75 |
| Munich LMU | 106 | 106 | 95 |
| Munich TU | 35 | 35 | 35 |
| Tübingen | 47 | 47 | 33 |
